# Illness presentation and quality of life in myalgic encephalomyelitis/chronic fatigue syndrome and post COVID-19 condition: a pilot Australian cross-sectional study

**DOI:** 10.1007/s11136-024-03710-3

**Published:** 2024-07-03

**Authors:** Breanna Weigel, Natalie Eaton-Fitch, Kiran Thapaliya, Sonya Marshall-Gradisnik

**Affiliations:** 1 National Centre for Neuroimmunology and Emerging Diseases, Griffith University, Gold Coast, QLD 4222 Australia; 2 Consortium Health International for Myalgic Encephalomyelitis, Griffith University, Gold Coast, QLD 4222 Australia; 3 School of Pharmacy and Medical Sciences, Griffith University, Gold Coast, QLD 4222 Australia

**Keywords:** Myalgic encephalomyelitis/chronic fatigue syndrome, Post COVID-19 condition, Post-Acute sequelae of COVID-19, Long COVID, Quality of life

## Abstract

**Purpose:**

Post COVID-19 Condition (PCC), being persistent COVID-19 symptoms, is reminiscent of Myalgic Encephalomyelitis/Chronic Fatigue Syndrome (ME/CFS)—a chronic multi-systemic illness characterised by neurocognitive, autonomic, endocrinological and immunological disturbances. This novel cross-sectional investigation aims to: (1) compare symptoms among people with ME/CFS (pwME/CFS) and people with PCC (pwPCC) to inform developing PCC diagnostic criteria; and (2) compare health outcomes between patients and people without acute or chronic illness (controls) to highlight the illness burdens of ME/CFS and PCC.

**Methods:**

Sociodemographic and health outcome data were collected from n = 61 pwME/CFS, n = 31 pwPCC and n = 54 controls via validated, self-administered questionnaires, including the 36-Item Short-Form Health Survey version 2 (SF-36v2) and World Health Organization Disability Assessment Schedule version 2.0 (WHODAS 2.0). PwME/CFS and pwPCC also provided self-reported severity and frequency of symptoms derived from the Canadian and International Consensus Criteria for ME/CFS and the World Health Organization case definition for PCC.

**Results:**

Both illness cohorts similarly experienced key ME/CFS symptoms. Few differences in symptoms were observed, with memory disturbances, muscle weakness, lymphadenopathy and nausea more prevalent, light-headedness more severe, unrefreshed sleep more frequent, and heart palpitations less frequent among pwME/CFS (all p < 0.05). The ME/CFS and PCC participants’ SF-36v2 or WHODAS 2.0 scores were comparable (all p > 0.05); however, both cohorts returned significantly lower scores in all SF-36v2 and WHODAS 2.0 domains when compared with controls (all p < 0.001).

**Conclusion:**

This Australian-first investigation demonstrates the congruent and debilitating nature of ME/CFS and PCC, thereby emphasising the need for multidisciplinary care to maximise patient health outcomes.

**Supplementary Information:**

The online version contains supplementary material available at 10.1007/s11136-024-03710-3.

## Plain English summary

Myalgic Encephalomyelitis/Chronic Fatigue Syndrome (ME/CFS) and Post COVID-19 Condition (PCC) are chronic illnesses that affect multiple body systems. While the exact biological causes of these illnesses are not yet known, people with ME/CFS (pwME/CFS) and people with PCC (pwPCC) experience similar symptoms and difficulties in daily functioning.

However, specific similarities and differences, which may warrant tailored approaches to management, have not yet been investigated in detail. Additionally, poor recognition of the impacts of these illnesses has prevented pwME/CFS and pwPCC from accessing necessary care. As the first comprehensive investigation of the illness experiences of pwME/CFS and pwPCC in Australia, this study serves to provide evidence to inform care pathways and changes to health policies, thereby ensuring that care aligns with patients’ needs.

Few differences were observed in the symptoms experienced by pwME/CFS and pwPCC in this study. Importantly, post-exertional malaise—the defining feature of ME/CFS—was equally as common, severe and frequent among pwPCC. The ME/CFS and PCC groups returned similar scores across all quality of life categories, indicating comparable effects on health and wellbeing. For both illness groups, all aspects of quality of life were considerably poorer when compared with people without acute or chronic illness.

Both ME/CFS and PCC have an extensive symptom burden and similar widespread impacts on patients’ lives. The findings of this study emphasise the importance of accessible, holistic care and support for both patient cohorts and highlight the potential role of existing protocols for ME/CFS in the diagnosis and management of PCC.

## Introduction

Post COVID-19 Condition (PCC), also known as Long COVID or Post-Acute Sequelae of COVID-19, is characterised by the persistence or new onset of symptoms among people with a history of Severe Acute Respiratory Syndrome Coronavirus-2 (SARS-CoV-2) infection [1–3]. The illness presentation of PCC has remarkable overlaps with that of Myalgic Encephalomyelitis/Chronic Fatigue Syndrome (ME/CFS) [3–10], which is a chronic multi-systemic illness associated with disruptions to neurocognitive, autonomic, endocrinological and immunological functioning [8, 11–13].

The hallmark feature of ME/CFS is post-exertional malaise, being the exacerbation of symptoms following physical, mental, or emotional effort [8, 12, 14]. Other key ME/CFS symptoms include cognitive dysfunction (commonly known as ‘brain fog’), sleep disturbances, pain, flu-like symptoms, cardiac dysrhythmias, gastrointestinal upset and thermostatic dysregulation [8–13, 15, 16]. Approximately 70% of ME/CFS cases are predated by an infection [8, 17–19] and numerous pathogens diverse in their virulence mechanisms and prevalence within the community have been tenuously implicated in the development of the condition [12, 13, 15, 17, 20–23]. Post-infectious cases of ME/CFS are most frequently attributed to Epstein-Barr virus—the causative agent of mononucleosis or ‘glandular fever’ [18, 24–26]; however, other microbes for which ME/CFS is a potential post-infectious sequela include person-to-person transmissible viruses (such cytomegalovirus, coxsackieviruses, Human Herpes Virus-6 and SARS-CoV-1) and vector-borne viruses (such Ross River virus, West Nile virus and Dengue virus), as well as bacterial (such as *Coxiella burnetii* and *Borrelia burgdorferi*) and parasitic (such as *Giardia lamblia*) infections [11–13, 15, 17, 20–23]. Hence, the emergence of PCC following acute COVID-19 illness may have positioned SARS-CoV-2 as a novel potential trigger of ME/CFS [9, 15].

Additionally, similarities in clinical and physiological measures (such as ion channel dysfunction, altered connectivity between brain regions, neuroendocrine disturbances, endothelial dysfunction, postural instability and orthostatic intolerance [3, 15, 27–30]) have been identified among people with ME/CFS (pwME/CFS) and people with PCC (pwPCC). However, the pathophysiological mechanisms underpinning these illnesses remain incompletely defined and biomarkers to confirm diagnoses of ME/CFS or PCC in clinical practice are yet to be established [3, 8, 12, 31–34]. Consequently, identifying cases of ME/CFS or PCC relies solely on clinical diagnosis, which requires both exclusion of other possible diagnoses and an illness presentation consistent with case criteria [1, 8, 13, 31, 35].

A diagnosis of ME/CFS typically requires patients to fulfil the Canadian Consensus Criteria (CCC) [11] or International Consensus Criteria (ICC) [12], in which ME/CFS is considered when a specified number of symptoms from each symptom category are present. Numerous case definitions have been established to describe the clinical syndrome of persistent COVID-19 symptoms following the resolution of acute viral infection [36, 37], typically requiring (1) previous SARS-CoV-2 infection, which may be laboratory confirmed or clinically suspected; and (2) at least one symptom that is persistent or new in onset following acute COVID-19 illness [1, 35, 37]. However, there exists discrepancy between PCC case definitions in the minimum illness duration required, which ranges from 4 weeks to 3 months post-infection [36, 37].

Symptoms must persist for at least 12 weeks after acute illness to fulfil the case definition developed by the World Health Organization (WHO) for ‘Post COVID-19 Condition’ [1], the National Institutes for Health and Excellence for ‘Post-COVID-19 Syndrome’ [35], and the European Society for Microbiology and Infectious Disease for ‘Long COVID’ [37]. The WHO case definition [1] additionally requires symptoms to impact one’s daily life. However, due to their relatively broad and non-specific nature, established case definitions for PCC likely capture a collection of post-COVID-19 sequelae [33, 38] and are unable to distinguish between COVID-19 survivors with tissue-specific damage and those with multi-systemic post-viral illness akin to ME/CFS [3, 15, 33, 39].

The comparable symptomatology of ME/CFS and PCC has been discussed in the literature [3, 9, 10, 16, 40]; however, some differences in symptom presentation have been noted between the two cohorts. For instance, approximately 10% to 20% of people with persistent COVID-19 symptoms experience dysgeusia or dysosmia [6, 41–43], yet these are not typical components of the ME/CFS illness presentation [8, 11–13, 40]. Some observational studies have also reported a lower burden of gastrointestinal disturbances [19, 44, 45], as well as a higher burden of respiratory difficulty [19, 46], among pwPCC when compared with pwME/CFS. However, there are considerable disparities between studies regarding the presentation of pain, flu-like symptoms, thermoregulation and cardiovascular symptoms among pwPCC compared with pwME/CFS [19, 30, 44–47]. Nevertheless, key ME/CFS symptoms, such as cognitive dysfunction, sleep disturbances and impairments in energy, appear to be similarly experienced among pwPCC [19, 30, 44–47].

Such resemblance in the clinical presentation, as well as the aetiology and physiological findings, of these two illnesses portend the potential role of ME/CFS in the illness progression of PCC [9, 10, 15]. This has important implications for refining PCC diagnostic criteria, in which guidelines informed by existing approaches to ME/CFS diagnosis may aid in identifying patients at risk of long-term illness and thereby facilitate early interventions to maximise patient health outcomes [9, 16, 26]. The present, exploratory investigation therefore aims to provide further insight into the illness presentation of PCC through detailed comparisons of symptom presentation among pwME/CFS and pwPCC.

In the existing literature, stringent case criteria to ascertain cohorts of pwME/CFS and pwPCC are not consistently employed to reduce potential confounding due to other medical conditions [19, 30, 44–47]. Additionally, published illness presentation data largely pertains to prevalence and symptoms are often compared in clusters rather than individually [30, 44, 46, 47]. This is the first study to directly compare symptom prevalence, severity and frequency among patients that fulfil stringent case criteria, including pwME/CFS meeting the CCC [11] or ICC [12] and pwPCC meeting the WHO case definition [1]. Such data is necessary for identifying similarities between these two illnesses, as well as understanding nuanced differences, to further develop PCC diagnostic criteria and tailored care protocols.

Considering these two illness cohorts in parallel also has important implications for care and policy due to their congruency in patients’ illness experiences [3, 16, 48]. While pharmacological interventions to manage ME/CFS and PCC symptoms have been proposed, there does not currently exist a universal curative therapy or pharmacological treatment capable of counteracting the pathophysiology of ME/CFS or PCC [3, 8, 13, 35]. Consequently, both illnesses require integrated, multidisciplinary management approaches to mitigate symptoms and impacts on life [8, 13, 35, 48].

Significantly compromised patient-reported health outcomes, including poor quality of life (QoL) and reduced functional capacity, have been reported among both pwME/CFS and pwPCC when compared with people without acute or chronic illness [49–55]. Research has suggested that impairments in health outcomes may persist among people reporting PCC recovery [56]; however, there is a paucity of data among recovered PCC cohorts and the findings in the existing literature are inconclusive [57]. All domains of QoL and functional capacity are affected by ME/CFS and PCC and, importantly, both illnesses considerably disrupt patients’ ability to perform typical activities of daily and working life [51–53, 58–60]. Such limitations in functioning range from a reduced capacity to maintain employment to an inability to independently perform self-care activities [8, 31, 61–63]. Hence, access to assistance from disability and social support services is paramount for pwME/CFS and pwPCC [61, 63–66]. However, pwME/CFS and pwPCC commonly face challenges in accessing necessary care and support both internationally and in the Australian context [61, 63, 64, 67–69].

In Australia, pwME/CFS and pwPCC are often deemed ineligible for necessary services such as income support or care subsidies [61, 64, 70]. To elucidate the impacts of these illnesses on patients’ lives and thereby provide evidence for improved care and support accessibility, this Australian-first investigation aims to compare the QoL and functional capacity of pwME/CFS and pwPCC with people without acute or chronic illness (controls). Poorer QoL and functional capacity scores across all domains were anticipated among the pwME/CFS and pwPCC when compared with controls. Hence, this study serves to inform changes to Australian healthcare policy that facilitate the provision of adequate and appropriate care and support to foster the best possible health outcomes for Australians living with these chronic multi-systemic illnesses.

## Materials and methods

### Study setting

This pilot cross-sectional study was conducted at the National Centre for Neuroimmunology and Emerging Diseases (NCNED) on the Gold Coast, Queensland, Australia from 1st March 2021 and 31st August 2022. The sample population for this study was obtained from the NCNED’s participant database, which includes pwME/CFS, pwPCC and controls who have responded to study advertisements distributed via the research centre’s newsletters, social media posts and collaborating physicians. Upon enrolment, all participants in the NCNED’s database completed the centre’s Research Registry Questionnaire—a self-administered survey, which has been described previously [71]. LimeSurvey (Carsten, Schmitz, Hamburg, Germany) [72] was employed to distribute the questionnaire to study participants online. Completed questionnaires were screened to determine participants’ eligibility for the present study.

This research was approved by the Griffith University Human Research Ethics Committee (HREC) (Reference Number: 2019/1005) and the Gold Coast University Hospital HREC (Reference Number: HREC/2019/QGC/56469), and has been conducted in accordance with the Australian Government National Health and Medical Research Council National Statement on Ethical Conduct in Human Research 2007 (updated 2018) [73] and the World Medical Association Declaration of Helsinki [74]. The present study also adheres to the Strengthening the Reporting of Observational Studies in Epidemiology Statement guidelines (S1 Table, Online Resource 1) [75].

### Study participants

Informed consent was electronically obtained from all study participants prior to their participation. Participants’ anamnesis (including any previous and current or active illnesses, injuries or surgeries) was collected to confirm their illness status (either a person with ME/CFS, a person with PCC or control), as well as to identify any comorbid manifestations or exclusionary diagnoses. Controls were defined in this study as those who did not report a formal diagnosis of any chronic health condition and had no evidence of underlying illness. PwPCC were required to fulfil the WHO definition [1] for PCC. Additional criteria for pwME/CFS included: (1) currently fulfilling or having a history of fulfilling at least one of the CCC [11] or ICC [12] for ME/CFS, (2) having had received a formal diagnosis of ME/CFS from a physician, and (3) not reporting a history of acute COVID-19 illness prior to ME/CFS onset. To ensure that the findings of the present study are appropriately attributed to ME/CFS and PCC and are not confounded by comorbid or pre-existing medical conditions, reported history of other formally diagnosed disease pathologies (such as genetic, metabolic, immunological (including autoimmune disease), neurological, cardiovascular, or respiratory disease), malignancy within the last 5 years, and formally diagnosed mental illness, chronic multi-systemic illness, or other post-viral illness were considered exclusionary. Respondents with concurrent or subsequent diagnoses of anxiety, depression, or overlapping chronic pain conditions captured within ME/CFS diagnostic criteria (such as fibromyalgia and irritable bowel syndrome) were not excluded [8, 11, 12].

### Sociodemographic characteristics

The following sociodemographic information was requested from all study participants via the Research Registry Questionnaire: age, sex assigned at birth, body mass index (BMI), Australian state of residence, highest level of education completed, employment status and (for participants who were not employed at the time of completing the questionnaire) whether illness or disability was responsible for unemployment.

### Illness characteristics and symptom presentation

All pwME/CFS and pwPCC completed the Research Registry Questionnaire and provided their illness duration, as well as symptom presentation (including symptom presence, severity and frequency) within the month prior to completing the questionnaire. Illness duration was calculated from the month and year in which participants reported first experiencing symptoms of their chronic multi-systemic illness. The symptoms assessed were derived from the CCC [11] and ICC [12] and align with the manifestations within the WHO case definition [1] for PCC. Hence, the questionnaire captured participants’ self-perceived experiences of post-exertional malaise, as well as symptoms from six major symptom categories, including: (1) Cognitive disturbances (impaired concentration and short-term memory loss); (2) Pain (headache, myalgia, arthralgia (without redness or swelling) and abdominal pain); (3) Sleep (sleep disturbances, such as insomnia, prolonged sleep (including naps), frequent awakenings, vivid dreams or nightmares and unrefreshed sleep); (4) Neurosensory, perceptual and motor disturbances (photophobia, sensitivity to noise or vibration, sensitivity to odour or taste and muscle weakness); (5) Immune, gastrointestinal and urinary disturbances (lymphadenopathy, laryngitis, nausea, bloating, altered bowel habits, such as diarrhoea and constipation, and urinary frequency or urinary urgency); and (6) Other autonomic manifestations (heart palpitations, light-headedness or dizziness, dyspnoea, sweating episodes, recurrent feelings of feverishness and cold extremities).

Symptom presence was defined as both: (1) having been experienced at least ‘a little of the time’ and at least at a ‘very mild’ level of severity; and (2) having been reported by the participant as being attributable to their chronic multi-systemic illness. PwME/CFS and pwPCC were subsequently categorised based on the most stringent ME/CFS case definition met according to their symptom presentation. Symptom severity and frequency were quantified on the five-point Likert scales employed in the 2005 Centers for Disease Control and Prevention’s Symptom Inventory Questionnaire for ME/CFS [76]. Symptom severity was measured as: (1) very mild, (2) mild, (3) moderate, (4) severe or (5) very severe, and symptom frequency as: (1) a little of the time, (2) some of the time, (3) a good bit of the time, (4) most of the time or (5) all the time.

### Patient-reported outcome measures

Five validated patient-reported outcome measures (PROMs) were employed in this study to capture the participants’ QoL and functional capacity. All pwME/CFS, pwPCC and controls completed the 36-Item Short-Form Health Survey version 2 (SF-36v2) [77] and the World Health Organization Disability Assessment Schedule version 2.0 (WHODAS 2.0) [78].

### SF-36v2

The SF-36v2 [77] assesses QoL across eight domains, including Physical Functioning, Role Limitations due to Physical Health Problems (also known as Role Physical), Bodily Pain, General Health Perceptions, Vitality, Social Functioning, Role Limitations due to Personal or Emotional Problems (also known as Role Emotional), and General Mental Health. Domain scores were calculated according to the scoring instructions in the version 2 update [77].

### WHODAS 2.0

The WHODAS 2.0 [78] quantifies functional capacity across seven domains: (1) Cognition, (2) Mobility, (3) Self-Care, (4) Getting Along, (5) Life Activities 1—general, (6) Life Activities 2—work and school, and (7) Participation. Given that many pwME/CFS are unable to work due to their illness, the Life Activities 2 items were omitted from the analysis. Weighted domain subscale scores were generated as outlined in the WHODAS 2.0 manual [78].

### Secondary PROMs

Participants were also invited to complete a second self-administered questionnaire comprising three additional validated PROMs for health and wellbeing, including the Hospital Anxiety and Depression Scale (HADS) [79], the Modified Fatigue Impact Scale (MFIS) [80], and Dr Bell’s Chronic Fatigue and Immune Dysfunction Syndrome (CFIDS) Disability Scale [81]. In addition to the Research Registry Questionnaire, the secondary PROMs questionnaire was completed by n = 29 pwME/CFS, n = 14 pwPCC and n = 8 controls.

### Statistical methods

Data analysis was performed with Statistical Package for the Social Sciences version 29 (IBM Corp, Armonk, New York) [82]. The statistical tests performed and reporting of results align with the Statistical Analysis and Methods in the Published Literature guidelines [83]. For continuous data, homogeneity of variances was assessed with Levene’s test and normality was investigated with Shapiro–Wilk (if n < 50) and Kolmogorov–Smirnov (if n ≥ 50) tests. The α-level for all statistical analyses was 0.05 and the results of any post-hoc analyses presented are p < 0.05 after correction for multiple comparisons. All p-values are provided to two significant figures except where p < 0.001. The number and percentage of participants with missing data are reported for each variable where applicable.

Categorical variables were compared between the study cohorts with Chi-square, Fisher-Freeman-Halton and Fisher’s exact tests. Post-hoc analyses for categorical variables were performed using pairwise comparisons and the Benjamini-Hochberg correction. Non-normally distributed continuous variables were compared between the study cohorts using Mann-Whitney *U* and Kruskal-Wallis *H* tests, with Dunn-Bonferroni post-hoc analyses and the Benjamini-Hochberg correction employed where applicable. To compare the non-parametric QoL scores of the cohorts while controlling for age and sex (as well as illness duration, where applicable), partial rank correlations were generated for the three possible pairings of the study cohorts and were subsequently adjusted for multiple comparisons with the Benjamini-Hochberg correction. For all relevant QoL comparisons, confounding variables were investigated using linear regression models and, where applicable, were analysed as covariates to confirm the robustness of the results. Reliability statistics were generated for all subscales of the QoL PROMs (except for Dr Bell’s CFIDS Disability Scale, as this PROM is a single-item measure). For each subscale, internal consistency was evaluated with McDonald’s ω. Sufficient internal consistency was defined as ω ≥ 0.7 [84].

## Results

The NCNED’s participant database (n = 1200) was screened for eligible study participants. Of the database participants, 250 people were deemed eligible. Data were available for n = 146 participants who had provided informed consent for prospective studies, including n = 61 pwME/CFS, n = 31 pwPCC and n = 54 controls.

### Sociodemographic characteristics

The sociodemographic data of the three participant cohorts are provided in Table 1. Most participants were female (78.7%, n = 48 pwME/CFS; 64.5%, n = 20 pwPCC; and 68.5%, n = 37 controls), resided in Queensland (72.1%, n = 44 pwME/CFS; 83.9%, n = 26 pwPCC; and 77.8%, n = 42 controls), and had completed tertiary education (55.7%, n = 34 pwME/CFS; 61.3%, n = 19 pwPCC; and 66.7%, n = 36 controls). Age was significantly higher among pwPCC (median (M) = 47.00, quartile 1 to 3 (Q1–3) = 39.00–56.00, 95% confidence interval (95% CI) = 41.00–54.00) compared with the ME/CFS (M = 42.00, Q1–3 = 30.15–52.20, 95% CI = 36.00–47.00) and control (M = 42.50, Q1–3 = 25.85–52.00, 95% = CI 34.00–47.00) cohorts (p < 0.05 after corrections). Significantly more pwME/CFS participants were unemployed (60.7%, n = 37) compared with the PCC (6.5%, n = 2) and control (9.3%, n = 5) groups (p < 0.05 after corrections). Unemployment was attributed to illness by 94.6% (n = 35) and 100.0% (n = 2) of the unemployed pwME/CFS and pwPCC, respectively.

**Table 1:** Sociodemographic characteristics of all study participants

	PwME/CFS (n = 61)	PwPCC (n = 31)	Controls (n = 54)	Test statistic	p
Age (years, M (Q1–3) [95% CI])	42.00 (30.15–52.20) [36.00–47.00]	47.00 (39.00–56.00) [41.00–54.00]	42.50 (25.85–52.00) [34.00–47.00]	6.679^a^	**0.035**^c,d^
Missing	0 (0.0)	0 (0.0)	0 (0.0)		
Sex (n (%))				2.612^b^	0.28
Female	48 (78.7)	20 (64.5)	37 (68.5)		
Male	13 (21.3)	11 (35.5)	17 (31.5)		
Other	0 (0.0)	0 (0.0)	0 (0.0)		
Missing	0 (0.0)	0 (0.0)	0 (0.0)		
BMI (M (Q1–3) [95% CI])	24.20 (21.20–27.30) [22.60–25.70]	26.80 (22.10–29.00) [22.70–28.10]	23.80 (21.55–26.40) [22.30–25.00]	5.151^a^	0.076
Missing	0 (0.0)	0 (0.0)	1 (1.9)		
State of residence (n (%))				7.180^b^	0.50
Australian Capital Territory	0 (0.0)	0 (0.0)	0 (0.0)		
New South Wales	8 (13.1)	1 (3.2)	5 (9.3)		
Northern Territory	0 (0.0)	0 (0.0)	0 (0.0)		
Queensland	44 (72.1)	26 (83.9)	42 (77.8)		
South Australia	0 (0.0)	0 (0.0)	0 (0.0)		
Tasmania	0 (0.0)	0 (0.0)	1 (1.9)		
Victoria	9 (14.8)	4 (12.9)	4 (7.4)		
Western Australia	0 (0.0)	0 (0.0)	1 (1.9)		
Missing	0 (0.0)	0 (0.0)	1 (1.9)		
Education (n (%))				10.024^b^	0.12
Primary school	0 (0.0)	0 (0.0)	0 (0.0)		
High school	16 (26.2)	6 (19.4)	9 (16.7)		
Undergraduate	15 (24.6)	15 (48.4)	15 (27.8)		
Postgraduate	19 (31.1)	4 (12.9)	21 (38.9)		
Other	11 (18.0)	6 (19.4)	8 (14.8)		
Missing	0 (0.0)	0 (0.0)	1 (1.9)		
Employment (n (%))				46.348^b^	** < 0.001**
Not employed	37 (60.7)	2 (6.5)	5 (9.3)		^e,f^
Reason for unemployment				20.138^b^	** < 0.001**
Illness	35 (94.6)	2 (100.0)	0 (0.0)		^f^
Other	2 (5.4)	0 (0.0)	5 (100.0)		^g^
Employed	24 (39.3)	29 (93.5)	48 (88.9)		^d,g^
Employment status				19.123^b^	** < 0.001**
Casual	8 (33.3)	1 (3.4)	6 (12.5)		^e^
Part-time	10 (41.7)	5 (17.2)	9 (18.8)		
Full-time	6 (25.0)	23 (79.3)	33 (68.8)		^d,g^
Missing	0 (0.0)	0 (0.0)	1 (1.9)		

*95% CI* 95% confidence interval, *BMI* body mass index, *M* median, *PwME/CFS* people with myalgic encephalomyelitis/chronic fatigue syndrome, *PwPCC* people post COVID-19 condition, *Q1–3* quartile 1 to 3

Bolded p-values indicate significance (p < 0.05)

^a^Kruskal-Wallis *H* statistic. ^b^Fisher-Freeman-Halton exact test statistic. ^c^PwPCC > Controls. ^d^PwPCC > PwME/CFS. ^e^PwME/CFS > PwPCC. ^f^PwME/CFS > Controls. ^g^Controls > PwME/CFS

### Illness characteristics

The illness characteristics of the pwME/CFS and pwPCC are summarised in Table 2. Most pwME/CFS fulfilled the ICC [12] (65.6%, n = 40), while 32.8% (n = 20) fulfilled the CCC [11]. One participant (1.6%) fulfilling the Fukuda criteria [85]—an earlier case definition for ME/CFS broader than the CCC [11] and ICC [12]—at the time of completing the questionnaire was included in the present study due to a history of meeting more stringent criteria and fluctuating symptoms. All pwPCC (100.0%, n = 31) met the WHO case definition [1] for PCC, with 58.1% (n = 18) also fulfilling at least one of the three diagnostic criteria for ME/CFS.

**Table 2:** Illness characteristics of and diagnostic criteria met by all study participants

	PwME/CFS (n = 61)	PwPCC (n = 31)	Controls (n = 54)	Test statistic	p
Total number of symptoms (M (Q1–3) [95% CI])	18 (15–20) [17–19]	14 (12–17) [12–16]	NA	461.00^a^	** < 0.001**
Missing	0 (0.0)	1 (3.2)			
ME/CFS criteria (n (%))^b^				7.328^c^	**0.019**
None	0 (0.0)	13 (41.9)	54 (100.0)		
Fukuda [85]	1 (1.6)	3 (9.7)	0 (0.0)		
CCC [11]	20 (32.8)	8 (25.8)	0 (0.0)		
ICC [12]	40 (65.6)	7 (22.6)	0 (0.0)		
Missing	0 (0.0)	0 (0.0)	0 (0.0)		
PCC criteria (WHO case definition [1], n (%))	0 (0.0)	31 (100.0)	0 (0.0)	NA	NA
Missing	0 (0.0)	0 (0.0)	0 (0.0)		
Illness duration (years, M (Q1–3) [95% CI])	10.00 (5.00–18.00) [8.00–13.00]	0.33 (0.25–0.60) [0.25–0.42]	NA	59.50^a^	** < 0.001**
Missing	0 (0.0)	1 (3.2)	NA		
HADS Anxiety (n (%))^d^				5.716^c^	0.20
Non-cases	16 (55.2)	7 (50.0)	8 (100.0)		
Mild or potential cases	7 (24.1)	3 (21.4)	0 (0.0)		
Moderate or probable cases	6 (20.7)	4 (28.6)	0 (0.0)		
Missing	0 (0.0)	0 (0.0)	0 (0.0)		
HADS Depression (n (%))^d^				8.034^c^	0.071
Non-cases	15 (51.7)	5 (35.7)	8 (100.0)		
Mild or potential cases	8 (27.6)	5 (35.7)	0 (0.0)		
Moderate or probable cases	6 (20.7)	4 (28.4)	0 (0.0)		
Missing	0 (0.0)	0 (0.0)	0 (0.0)		

*95% CI* 95% confidence interval, *CCC* Canadian Consensus Criteria, *HADS* hospital anxiety and depression scale, *ICC* International Consensus Criteria, *M* median, *NA* not applicable, *PwME/CFS* people with myalgic encephalomyelitis/chronic fatigue syndrome, *PwPCC* people with post COVID-19 condition, *Q1–3* quartile 1 to 3, *WHO* World Health Organization

Bolded p-values indicate significance

^a^Mann-Whitney *U* statistic. ^b^Percentage of those fulfilling at least the Fukuda case definition. ^c^Fisher-Freeman-Halton exact test statistic. ^d^Values provided for n = 29 ME/CFS participants, n = 14 PCC participants and n = 8 controls

### Symptom presentation

**Fig. 1:**
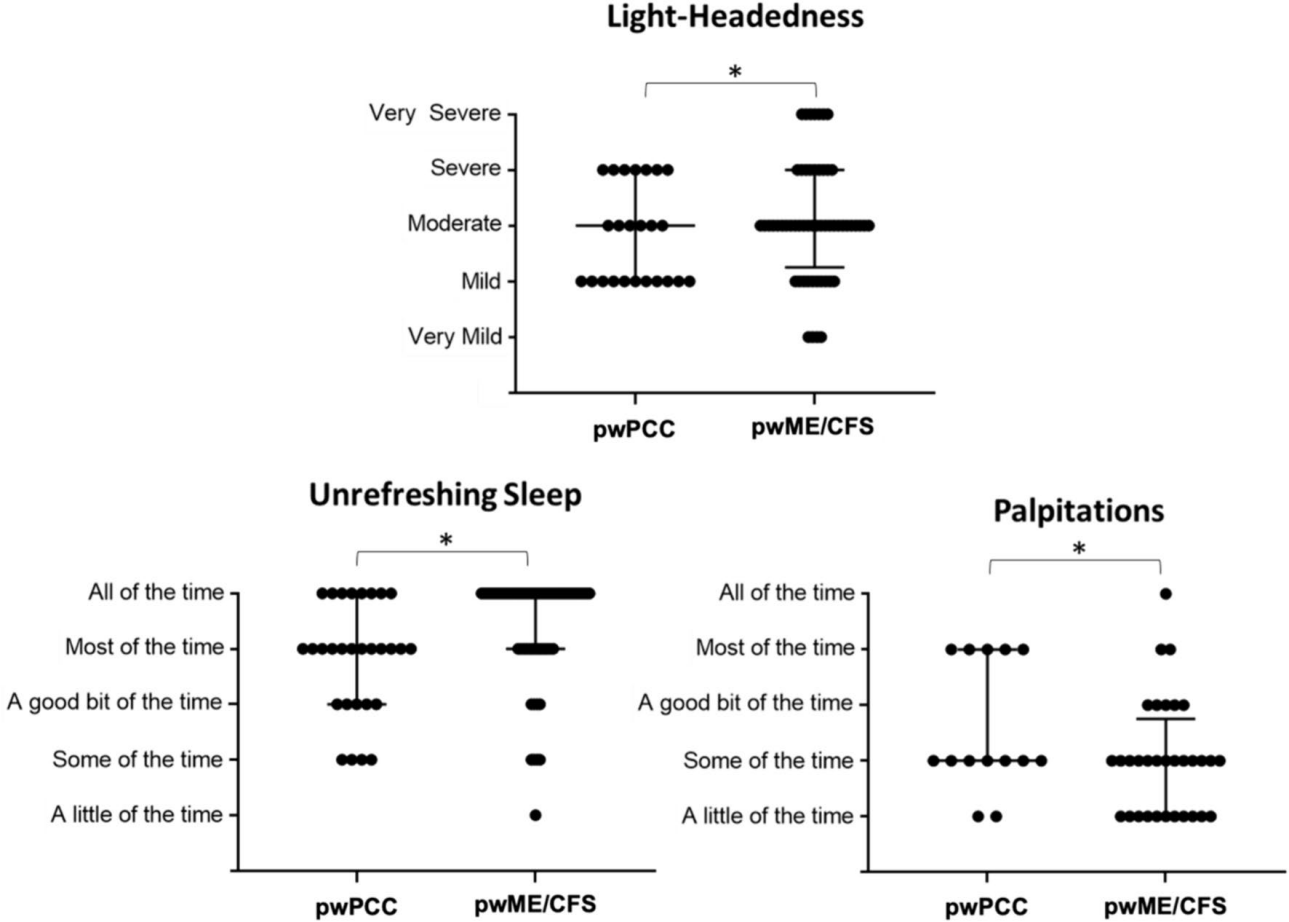
Severity and frequency for symptoms returning significance between the two illness cohorts. Figure generated with GraphPad. *PwME/CFS* people with myalgic encephalomyelitis/chronic fatigue syndrome, *PwPCC* People with Post COVID-19 Condition. Symptom severity and frequency data is provided for participants experiencing the symptom in question within the month prior to completing the questionnaire and attributing the symptom in question to their chronic multi-systemic illness. *p < 0·05

Table 3 presents the results of symptom prevalence, severity and frequency comparisons between the two illness cohorts. Complete symptom presentation data are summarised in S2 to S3 Tables, Online Resource 1. Severity and frequency distributions for symptoms that differed significantly between the two cohorts are presented in Fig. 1.

**Table 3:** Summary of symptom presentation comparisons between the ME/CFS and PCC participants

	PwME/CFS (n = 61)			PwPCC (p = 31)			p		
	Prevalence n (%)^a^	Severity M (C)^b,c^	Frequency M (C)^b,d^	Prevalence n (%)^a^	Severity M (C)^b,c^	Frequency M (C)^b,d^	Prevalence^a^	Severity^b,c^	Frequency^b,d^
Post-exertional malaise	61 (100.0)	4 (0.716)	5 (0.699)	30 (96.8)	4 (0.736)	4 (0.745)	NA	1.0	0.091
Cognitive disturbances									
Impaired concentration	61 (100.0)	3 (0.655)	4 (0.568)	29 (93.5)	3 (0.696)	3 (0.586)	0.33	0.52	0.63
Short-term memory loss	42 (68.9)	3 (0.705)	3 (0.656)	14 (45.2)	3 (0.773)	3–4 (0.589)	**0.039**	0.62	0.26
Pain									
Headache	56 (91.8)	3 (0.766)	3 (0.583)	25 (80.6)	3 (0.755)	3 (0.441)	0.29	0.64	0.050
Myalgia	56 (91.8)	3 (0.709)	4 (0.570)	23 (74.2)	3 (0.701)	3 (0.621)	0.055	0.42	0.45
Arthralgia (without redness or swelling)	42 (68.9)	3 (0.686)	3 (0.561)	15 (48.4)	3 (0.796)	3 (0.633)	0.11	0.26	0.84
Abdominal pain	39 (63.9)	2 (0.663)	2 (0.519)	14 (45.2)	2–3 (0.618)	2 (0.600)	0.17	0.83	0.43
Sleep									
Sleep disturbances^e^	57 (93.4)	3 (0.732)	3 (0.621)	26 (83.9)	3 (0.684)	3 (0.621)	0.43	0.64	0.64
Unrefreshed sleep	60 (98.4)	4 (0.723)	5 (0.643)	29 (93.5)	3 (0.693)	4 (0.657)	1.0	0.29	**0.011**
Neurosensory, perceptual and motor disturbances									
Photophobia	50 (82.0)	3 (0.748)	3 (0.624)	20 (64.5)	3 (0.734)	3 (0.648)	0.12	0.94	0.42
Sensitivity to noise or vibration	48 (78.7)	3 (0.666)	3 (0.557)	20 (64.5)	3 (0.669)	3 (0.459)	0.31	0.87	0.78
Sensitivity to odour or taste	29 (47.5)	3 (0.687)	3 (0.526)	10 (32.3)	2 (0.776)	2 (0.565)	0.26	0.24	0.67
Muscle weakness	53 (86.9)	3 (0.679)	3 (0.595)	15 (48.4)	3 (0.649)	3 (0.547)	** < 0.001**	0.33	0.32
Immune, gastrointestinal and urinary disturbances									
Lymphadenopathy	32 (52.5)	3 (0.771)	3 (0.598)	7 (22.6)	2 (0.811)	2 (0.556)	**0.013**	0.57	0.17
Laryngitis	39 (63.9)	2 (0.657)	3 (0.585)	17 (54.8)	2 (0.751)	3 (0.496)	0.65	1.0	0.67
Nausea	45 (73.8)	2 (0.727)	2 (0.611)	12 (38.7)	2 (0.666)	2 (0.709)	**0.0026**	0.26	1.0
Bloating	38 (62.3)	3 (0.621)	2 (0.476)	18 (58.1)	3 (0.600)	3 (0.576)	1.0	0.69	0.25
Altered bowel habits^f^	40 (65.6)	3 (0.595)	3 (0.390)	13 (41.9)	2 (0.608)	3 (0.425)	0.070	0.34	0.97
Urinary frequency or urinary urgency	23 (37.7)	3 (0.740)	3 (0.640)	7 (22.6)	3 (0.765)	3 (0.493)	0.24	0.16	0.43
Other autonomic manifestations									
Heart palpitations	32 (52.5)	2 (0.644)	2 (0.663)	14 (45.2)	3 (0.684)	2 (0.558)	0.66	0.13	**0.040**
Light-headedness or dizziness	56 (91.8)	3 (0.650)	3 (0.471)	24 (77.4)	3 (0.685)	2–3 (0.475)	0.17	**0.011**	0.28
Dyspnoea	36 (59.0)	2 (0.739)	2 (0.576)	18 (58.1)	3 (0.775)	3 (0.655)	1.0	0.37	0.17
Sweating episodes	27 (44.3)	3 (0.623)	2 (0.467)	8 (25.8)	3 (0.792)	2 (0.915)	0.12	0.87	0.28
Recurrent feelings of feverishness	23 (37.7)	3 (0.624)	2 (0.584)	11 (35.5)	3 (0.667)	2 (0.556)	1.0	0.95	0.90
Cold extremities	40 (65.6)	3 (0.603)	3 (0.522)	13 (41.9)	3 (0.826)	2 (0.694)	0.070	0.57	0.45

*C* consensus, *M* median, *NA* not applicable, *PwME/CFS* people with myalgic encephalomyelitis/chronic fatigue syndrome, *PwPCC* people with post COVID-19 condition

Bolded p-values indicate significance (p < 0.05)

^a^Self-reported and believed by participant to be attributable to either ME/CFS or PCC (depending on illness status). This does not include participants with missing data, nor does this include participants who stated that they, within the month prior to completing the questionnaire, did not experience the symptom in question, were unsure if they had experienced the symptom in question, had experienced the symptom in question but were unsure if the symptom was attributable to their chronic multi-systemic illness or had experienced the symptom in question but believed that the symptom was not attributable to their chronic multi-systemic illness (additional data, including the participants with missing data, is provided in S2 and S3 Tables, Online Resource 1). ^b^Among those who reported experiencing the symptom in question within the month prior to completing the questionnaire and believed the symptom to be attributable to their chronic multi-systemic illness. ^c^Likert scale values: (1) Very mild; (2) Mild; (3) Moderate; (4) Severe and (5) Very severe. ^d^Likert scale values: (1) A little of the time; (2) Some of the time; (3) A good bit of the time; (4) Most of the time; (5) All the time. ^e^Includes manifestations such as insomnia, prolonged sleep (including naps), frequent awakenings and vivid dreams or nightmares. ^f^Includes manifestations such as diarrhoea and constipation

PwME/CFS experienced a greater total number of symptoms than pwPCC, with median scores of 18 (Q1–3 = 15–20, 95% CI = 17–19) and 14 (Q1–3 = 12–17, 95% CI = 12–16), respectively (p < 0.001). However, hallmark ME/CFS symptoms (including post-exertional malaise, cognitive impairments and unrefreshed sleep) were comparable in prevalence, severity and frequency between the two illness cohorts, except that pwME/CFS were more likely to experience unrefreshed sleep ‘all the time’ (p < 0.05 after corrections). Post-exertional malaise was experienced by all pwME/CFS (100.0%, n = 61) and pwPCC (100.0%, n = 30) who provided valid symptom data. Additionally, impaired concentration was highly prevalent in both pwME/CFS and pwPCC (100.0%, n = 61 and 93.5%, n = 29, respectively, p = 0.33), as was unrefreshed sleep (98.4%, n = 60 and 93.5%, n = 29, respectively, p = 1.0). A median severity score of ‘severe’ was observed for post-exertional malaise among pwME/CFS (Consensus (C) = 0.716) and pwPCC (C = 0.736), as well as for unrefreshed sleep among pwME/CFS (C = 0.723). Post-exertional malaise and unrefreshed sleep had a median frequency rating of ‘all the time’ for pwME/CFS (C = 0.699 and C = 0.643, respectively) and ‘most of the time’ for pwPCC (C = 0.745 and C = 0.657, respectively).

Symptom presentation was largely comparable between the two cohorts, with few significant differences observed. Short-term memory loss, muscle weakness, lymphadenopathy and nausea were more prevalent among pwME/CFS (68.9%, n = 42; 86.9%, n = 53; 52.5%, n = 32; and 73.8%, n = 45, respectively) when compared with the PCC (45.2%, n = 14; 48.4%, n = 15; 22.6%, n = 7; and 38.7%, n = 12, respectively) cohort (p = 0.039, p < 0.001, p = 0.013 and p = 0.0026, respectively). Among the participants experiencing light-headedness or dizziness, pwPCC were more likely to be mildly affected than pwME/CFS (p < 0.05 after corrections). PwPCC were more likely to be affected by heart palpitations ‘most of the time’ than pwME/CFS experiencing this symptom within the month prior to completing the questionnaire (p < 0.05 after corrections).

### Quality of life and functional capacity

Summary statistics for all QoL PROMs are provided in Table 4. For all PROMs, poorer QoL scores were observed among pwME/CFS and pwPCC in every domain (except HADS Anxiety) when compared with controls (p < 0.001, uncorrected). PwME/CFS and pwPCC did not differ significantly in any domain of the QoL PROMs.

**Table 4:** Quality of life among all study participants

	PwME/CFS (n = 61)	PwPCC (n = 31)	Controls (n = 53)^b^	PwME/CFS v Controls^a^ (n = 114)		PwPCC v Controls^a^ (n = 84)		PwME/CFS v PwPCC^a^ (n = 92)	
				*r*	Uncorrected p	*r*	Uncorrected p	*r*	Uncorrected p
SF-36v2 (M (Q1–3) [95% CI])^c^									
Physical Functioning	30.00 (15.00–57.50) [20.00–45.00]	40.00 (15.00–75.00) [30.00–65.00]	100.00 (100.00–100.00) [100.0–100.00]	0.873	** < 0.001**	0.862	** < 0.001**	0.005	0.96
Role Physical	18.75 (0.00–31.25) [0.00–25.00]	12.50 (0.00–50.00) [0.00–31.25]	100.00 (100.00–100.00) [100.00–100.00]	0.899	** < 0.001**	0.904	** < 0.001**	0.005	0.97
Bodily Pain	45.00 (22.50–57.50) [32.50–45.00]	40.00 (22.50–57.50) [22.50–55.00]	90.00 (88.75–100.00) [90.00–100.00]	0.749	** < 0.001**	0.675	** < 0.001**	0.073	0.50
General Health	25.00 (16.67–39.59) [20.83–29.17]	41.67 (29.17–66.67) [29.17–58.33]	79.17 (68.75–85.42) [75.00–79.17]	0.851	** < 0.001**	0.648	** < 0.001**	0.427	0.26
Vitality	6.25 (0.00–18.75) [6.25–12.50]	12.50 (6.25–18.75) [6.25–18.75]	75.00 (62.50–75.00) [62.50–75.00]	0.856	** < 0.001**	0.815	** < 0.001**	0.030	0.78
Social Functioning	25.00 (0.00–37.50) [12.50–25.00]	25.00 (12.50–50.00) [12.50–37.50]	100.00 (100.00–100.00) [100.00–100.00]	0.870	** < 0.001**	0.877	** < 0.001**	0.074	0.50
Role Emotional	75.00 (33.33–95.84) [58.33–75.00]	50.00 (16.67–83.33) [25.00–75.00]	100.00 (91.67–100.00) [100.00–100.00]	0.550	** < 0.001**	0.635	** < 0.001**	0.020	0.85
Mental Health	60.00 (42.50–77.50) [55.00–70.00]	55.00 (35.00–75.00) [40.00–70.00]	85.00 (70.00–85.00) [75.00–85.00]	0.468	** < 0.001**	0.574	** < 0.001**	− 0.049	0.65
WHODAS 2.0 (M (Q1–3) [95% CI])^c^									
Cognition	50.00 (40.00–60.00) [50.00–55.00]	40.00 (20.00–55.00) [25.00–55.00]	0.00 (0.00–15.00) [0.00–10.00]	− 0.727	** < 0.001**	− 0.611	** < 0.001**	− 0.171	0.11
Mobility	56.25 (34.38–75.00) [43.75–68.75]	43.75 (18.75–56.25) [31.25–56.25]	0.00 (0.00–6.25) [0.00–0.00]	− 0.774	** < 0.001**	− 0.679	** < 0.001**	− 0.039	0.72
Self-Care	30.00 (10.00–50.00) [20.00–40.00]	0.00 (0.00–40.00) [0.00–30.00]	0.00 (0.00–0.00) [0.00–0.00]	− 0.696	** < 0.001**	− 0.474	** < 0.001**	− 0.069	0.52
Getting Along	50.00 (20.00–70.00) [40.00–50.00]	30.00 (10.00–50.00) [10.00–50.00]	0.00 (0.00–10.00) [0.00–10.00]	− 0.591	** < 0.001**	− 0.413	** < 0.001**	− 0.181	0.092
Life Activities 1	80.00 (50.00–100.00) [60.00–90.00]	70.00 (40.00–90.00) [40.00–90.00]	0.00 (0.00–20.00) [0.00–0.00]	− 0.778	** < 0.001**	− 0.714	** < 0.001**	− 0.094	0.38
Participation	62.50 (50.00–75.00) [54.17–70.83]	50.00 (29.17–62.50) [37.50–58.33]	0.00 (0.00–6.25) [0.00–4.17]	− 0.797	** < 0.001**	− 0.742	** < 0.001**	− 0.126	0.24
HADS (M (Q1–3) [95% CI])^d,e^									
Anxiety	7.00 (3.00–9.50) [4.00–9.00]	7.50 (4.00–12.50) [4.00–14.00]	3.50 (1.25–6.75) [0.00–7.00]	− 0.385	0.023	− 0.306	0.19	0.264	0.10
Depression	7.00 (4.00–10.00) [5.00–9.00]	8.00 (6.00–11.00) [6.00–11.00]	1.00 (0.00–1.00) [0.00–2.00]	− 0.713	** < 0.001**	− 0.794	** < 0.001**	0.289	0.070
MFIS (M (Q1–3) [95% CI])^d^									
Physical^f^	30.00 (25.50–31.00) [26.00–31.00]	30.00 (22.75–31.25) [22.00–32.00]	0.00 (0.00–5.50) [0.00–9.00]	− 0.713	** < 0.001**	− 0.760	** < 0.001**	− 0.172	0.29
Cognitive^g^	28.00 (23.00–33.50) [24.00–32.00]	27.50 (20.00–32.25) [20.00–33.00]	1.00 (1.00–5.75) [0.00–12.00]	− 0.705	** < 0.001**	− 0.809	** < 0.001**	0.025	0.88
Psychosocial^h^	5.00 (4.00–7.00) [5.00–7.00]	6.00 (4.75–7.00) [4.00–7.00]	0.00 (0.00–0.00) [0.00–2.00]	− 0.721	** < 0.001**	− 0.829	** < 0.001**	0.170	0.30
Dr Bell’s CFIDS Disability Scale (M (C))^d,i^	40.0% (0.791)	40.0% (0.746)	100.0% (0.968)	0.702	** < 0.001**	0.804	** < 0.001**	− 0.022	0.89

*95% CI* 95% confidence interval, *C* consensus, *CFIDS* chronic fatigue and immune dysfunction syndrome, *HADS* hospital anxiety and depression scale, *M* median, *MFIS* modified fatigue impact scale, *PwME/CFS* people with myalgic encephalomyelitis/chronic fatigue syndrome, *PwPCC* people with post COVID-19 condition, *Q1–3* quartile 1 to 3, *SF-36v2* 36-item short-form health survey (version 2), *WHODAS 2*.*0* World Health Organization disability assessment schedule (version 2.0)

Uncorrected p-values are reported for each partial correlation model. Bolded uncorrected p-values indicate significance (p < 0.05) after correction for multiple comparisons. Any changes in significance arising from robustness checks are reported

^a^Data available for n = 61 pwME/CFS, n = 31 pwPCC and n = 53 controls. Data missing for n = 1 control. All QoL PROM subscales were non-parametric and analysed with partial rank correlations controlling for age and sex (as well as illness duration for comparisons between the pwME/CFS and pwPCC). ^b^QoL PROM data was not provided for n = 1 control. ^c^Each domain has a minimum score of 0 and a maximum score of 100. ^d^Values provided for n = 29 pwME/CFS, n = 14 pwPCC and n = 8 controls. ^e^Each domain has a minimum score of 0 and a maximum score of 21. ^f^This domain has a minimum score of 0 and a maximum score of 36. ^g^This domain has a minimum score of 0 and a maximum score of 40. ^h^This domain has a minimum score of 0 and a maximum score of 8. ^i^Values presented are reflective of percentage total functioning with a minimum score of 0.0% and a maximum score of 100.0%

Complete reliability statistics for each subscale of the QoL PROMs are provided in S4 Table. McDonald’s ω was greater than 0.7 for most subscales among the ME/CFS, PCC and control participants, indicating sufficient internal consistency. Only the SF-36v2 General Health domain among pwME/CFS and the SF-36v2 Vitality domain among pwPCC returned ω values less than 0.7 (ω = 0.569 and ω = 0.504, respectively).

### SF-36v2

Vitality and Role Physical were associated with the lowest scores of all the SF-36v2 domains for both the ME/CFS (M = 6.25, Q1–3 = 0.00–18.75, 95% CI = 6.25–12.50 and M = 18.75, Q1–3 = 0.00–31.25, 95% CI = 0.00–25.00, respectively) and PCC (M = 12.50, Q1–3 = 6.25–18.75, 95% CI = 6.25–18.75 and M = 12.50, Q1–3 = 0.00–50.00, 95% = CI 0.00–31.25, respectively) participants. The Role Emotional and Mental Health domains of the SF-36v2 were the least impacted for both the ME/CFS (M = 75.00, Q1–3 = 33.33–95.84, 95% CI = 58.33–75.00 and M = 60.00, Q1–3 = 42.50–77.50, 95% CI = 55.00–70.00, respectively) and PCC (M = 50.00, Q1–3 = 16.67–83.33, 95% CI = 25.00–75.00 and M = 55.00, Q1–3 = 35.00–75.00, 95% CI = 40.00–70.00, respectively) cohorts.

### WHODAS 2.0

Life Activities 1 was associated with the greatest illness impact of all the WHODAS 2.0 domains among the ME/CFS (M = 80.00, Q1–3 = 50.00–100.00, 95% CI = 60.00–90.00) and PCC participants (M = 70.00, Q1–3 = 40.00–90.00, 95% CI = 40.00–90.00). Self-Care was the WHODAS 2.0 domain least impacted by the participants’ illness in both the ME/CFS (M = 30.00, Q1–3 = 10.00–50.00, 95% CI = 20.00–40.00) and PCC (M = 0.00, Q1–3 = 0.00–40.00, 95% CI = 0.00–30.00) groups.

### Secondary PROMs

Additional QoL and functional capacity PROMs (including the HADS, MFIS and Dr Bell’s CFIDS Disability Scale) were completed by n = 51 study participants (n = 29 pwME/CFS, n = 14 pwPCC and n = 8 controls). Higher HADS Depression scores were returned by pwME/CFS and pwPCC compared with controls (both p < 0.001, uncorrected). HADS Anxiety scores were comparable across the three participant cohorts. When compared with controls, functional capacity was impaired in the Physical, Cognitive and Psychosocial subscales of the MFIS among both the ME/CFS (all p < 0.001, uncorrected) and PCC (all p < 0.001, uncorrected) participants. All MFIS domain scores were comparable between the two illness cohorts. The Physical subscale returned the highest scores (relative to the maximum possible score) of all the MFIS domains among the ME/CFS (M = 30.00, Q1–3 = 25.50–31.00, 95% CI = 26.00–31.00) and PCC (M = 30.00, Q1–3 = 22.75–31.25, 95% CI = 22.00–32.00) cohorts. Both pwME/CFS and pwPCC returned a significantly lower median score of 40.0% for Dr Bell’s CFIDS Disability Scale (C = 0.791 and C = 0.746, respectively) compared with controls (both p < 0.001, uncorrected).

## Discussion

The present study shares novel, detailed comparisons of illness presentation among pwME/CFS and pwPCC fulfilling stringent diagnostic criteria and investigates the impacts of these illnesses on patients’ QoL and functional capacity when compared with controls. This vital investigation further characterises the illness presentation of PCC, identifying remarkable similarities of this emerging illness with ME/CFS. These findings provide insight into the clinical case definition of PCC, as well as the potential use of existing ME/CFS management approaches among pwPCC. Additionally, this publication documents the profound and widespread illness burden experienced by pwME/CFS and pwPCC in Australia, thereby necessitating health policy reforms that facilitate improved accessibility of necessary care and support services for Australians living with these illnesses.

The present study observed marginal differences in illness presentation between pwPCC and pwME/CFS, with notable similarities between the two cohorts in key symptoms typically experienced by pwME/CFS, such as post-exertional malaise, neurocognitive dysfunction and sleep disturbances. All pwME/CFS and pwPCC providing valid symptom data experienced post-exertional malaise in the current study. Vernon et al. and Twomey et al. reported a similar post-exertional malaise prevalence of 99 and 94.8%, respectively, among people experiencing ongoing COVID-19 symptoms [46, 86]. While a lower prevalence of post-exertional malaise among people with post-COVID-19 sequelae was observed by Bonilla et al. (82.8%) and Retornaz et al. (78%) [44, 87], the appearance of the hallmark symptom of ME/CFS among this novel illness cohort suggests that post-exertional malaise is a noteworthy component of the PCC illness presentation and should be considered in diagnostic criteria and care provision.

Like the present investigation, international studies have similarly reported a comparable prevalence of key ME/CFS symptoms among people with persistent COVID-19 symptoms [19, 44–47, 86–88]. Among the cardinal ME/CFS symptoms, only memory loss was significantly more prevalent (p = 0.039) and unrefreshed sleep significantly more frequent (p = 0.011) among pwME/CFS than pwPCC in the present study. However, this may be explained by 51.6% of PCC participants not fulfilling stringent ME/CFS case criteria, as Kedor et al. observed comparable prevalence and severity of memory problems and sleep disturbances among pwME/CFS and pwPCC meeting the CCC [47].

The few remaining significant differences in symptom presentation between the cohorts included a higher prevalence of muscle weakness (p < 0.001), lymphadenopathy (p = 0.013) and nausea (p = 0.0026), greater severity of light-headedness (p = 0.011), and reduced frequency of heart palpitations (p = 0.0400) among pwME/CFS. However, the existing literature is incongruous regarding the presentation of such symptoms among pwME/CFS and pwPCC [19, 30, 44–47]. Additionally, comparisons of illness presentation have largely focused on symptom prevalence and few investigations have compared symptom frequency and severity among these two illness cohorts. Publications reporting such data among pwME/CFS and pwPCC have combined symptom prevalence within measures of severity and frequency [19, 45], which may overestimate differences in illness presentation.

Like the present investigation, Kedor et al. reported marginal differences in symptom severity among people with post-COVID-19 sequelae (including both those fulfilling and not fulfilling the CCC [11] for ME/CFS) and pwME/CFS [47]. Most symptoms were comparable in presentation in a study by Azcue et al. among people meeting the WHO case definition [1] for PCC and pwME/CFS; however, differences were observed in the severity and frequency of pain, thermostatic, neurosensory and gastrointestinal symptoms, as well as weight changes and unrefreshed sleep [45]. Unlike the present investigation, Jason et al. reported significant differences in the severity of most symptoms, except neurocognitive disturbances, among people with self-reported persistent COVID-19 symptoms (without a minimum illness duration threshold) and pwME/CFS [19]. Hence, the overlap of key ME/CFS symptoms, in addition to the disparity in the literature regarding the presentation of accessory symptoms, foregrounds the importance of developing PCC diagnostic criteria capable of delineating illness subtypes, including those with ME/CFS-like illness, with sufficient specificity.

In the present study, 58.1% of pwPCC fulfilled ME/CFS criteria. The prevalence of ME/CFS among people with persistent COVID-19 symptoms was reported by Kedor et al. and Bonilla et al. as 45.2 and 43%, respectively [47, 87]. Other studies have observed considerably lower proportions of people with ongoing COVID-19 symptoms fulfilling ME/CFS criteria, with the prevalence of ME/CFS reported as 16.8% by Tokumasu et al. and 8.1% by AlMuhaissen et al. [88, 89]. However, this may be due to participants in these studies having an illness duration of less than 6 months (a minimum illness duration threshold required by many ME/CFS case definitions [90]), in which Aly et al. observed that 53% of their study population would have met ME/CFS criteria had illness duration requirements been met [91]. Hence, fulfilment of ME/CFS criteria following acute COVID-19 illness may be an indicator of illness trajectory and a means of identifying pwPCC at risk of long-term illness.

While observational studies have reported recovery within 12 months in 40 to 50% of people with persistent COVID-19 symptoms [92, 93], the long-term health outcomes of PCC are not yet known [3]. Nevertheless, the findings of the present study underscore the risk of developing permanent chronic illness and disability following acute COVID-19 illness and foreground the potential role of ME/CFS in the illness progression and diagnosis of PCC. This also has important ramifications for estimating the future healthcare burdens of post-COVID-19 sequelae (as over 90% of people with ME/CFS experience life-long illness [18, 94]) and emphasises the importance of reducing SARS-CoV-2 transmission [95, 96].

The analogous QoL and functional capacity observed among pwME/CFS and pwPCC (in both the present and existing publications [27, 47]) also highlights the profound and widespread impact of this novel illness and validates the illness experiences of pwPCC, who continue to face stigma [63, 97, 98]. This publication echoes existing findings that ME/CFS and PCC have a noteworthy impact on one’s ability to perform daily activities due to physical functional limitations [51–53, 58–60], with the SF-36v2 Role Physical, WHODAS 2.0 Life Activities 1, WHODAS 2.0 Participation and MFIS Physical scores among the poorest of their corresponding PROMs. SF-36v2 Vitality scores were also notably poorer among pwME/CFS and pwPCC when compared with controls, indicating considerable impairments in energy interfering with life activities.

These findings exemplify the physically disabling nature of ME/CFS and PCC, which must be considered in the provision of care for people with these illnesses. The congruent impairments in QoL and functional capacity are also of note due to the significantly shorter illness duration of PCC when compared with ME/CFS. Importantly, the impacts on daily activities observed in this study emphasise the need for integrated approaches across the healthcare, disability and social support sectors for pwME/CFS and pwPCC in Australia [35, 61, 64, 66]. Access of disability and social support services has long been an arduous process for Australians with ME/CFS and, more recently, for those with PCC, with neither illness currently recognised as a disability in Australian health policy [61, 64]. These findings, combined with the high prevalence of post-exertional malaise, also have implications for clinical practice, reinforcing the importance of pacing strategies and engagement of allied health professionals, such as occupational therapists, in the management of ME/CFS, as well as PCC [3, 8, 13, 31, 32].

Mental health outcomes, including the SF-36v2 Role Emotional, SF-36v2 Mental Health and HADS Depression subscales, were significantly impacted among pwME/CFS and pwPCC when compared with controls in the present study. However, it is worth noting that mental health outcomes were the least affected of the QoL PROM domains and that these impacts are likely secondary to living with chronic illness and are not the primary cause of either ME/CFS or PCC [8]. In the present study, the HADS Anxiety subscale was the only QoL PROM domain that was not significantly different among the pwME/CFS and pwPCC when compared with controls. There was also no significant difference in the distribution of potential or probable anxiety or depression cases (determined via the HADS) among the three study cohorts. However, as the HADS was a component of the secondary PROM questionnaire, which was completed by a subset of the total study population, the lack of significance in the HADS subscales may be due to the smaller sample size.

### Strengths and limitations

It should be noted that the present cross-sectional study captured data at a single point in time. Detailed longitudinal data using a larger sample size is paramount to understand the relationship between PCC symptoms at onset, including ME/CFS-like illness presentation, and prognosis. Future longitudinal research should also correlate the trajectory of symptoms with the pathologies that have been identified among the two illnesses. Additionally, comparisons between pwPCC and pwME/CFS who have a comparable illness duration are warranted to determine whether the differences in symptom presentation are illness- or time-dependent [19].

The potential for volunteer bias in the present study must be acknowledged, as participants provided questionnaire data when participating in other projects at the NCNED research centre [27, 28, 99–101]. Some pwPCC providing symptom and health outcome data earlier in their illness progression prospectively fulfilled the WHO case definition [1] and therefore were still deemed eligible for inclusion in the present study. This current investigation also utilises self-reported data and, as the survey responses are subjective, participants may not assess themselves similarly. Employment data should be interpreted with caution, as most pwPCC who reported being in full-time employment stated that they were on sick leave or organised reduced working hours. Finally, the study cohorts may underrepresent the illness experiences of people from marginalised populations. Future inclusive research is required to inform tailored approaches to care and support for people belonging to marginalised populations who live with ME/CFS or PCC in Australia.

Importantly, this investigation serves as a pilot study providing novel patient experience data to inform Australian healthcare policy. Validated diagnostic criteria were employed to ascertain the study cohorts, including the CCC [11] and ICC [12] for ME/CFS and the WHO case definition [1] for PCC. These diagnostic criteria are the most stringent criteria available and, for this reason, were employed to ensure people with other chronic illnesses were not selected into the study sample [13, 102]. The use of validated measures of symptom presentation and QoL also mitigated the potential for information bias.

## Conclusions

The manifestations of ME/CFS and PCC are remarkably similar, with marginal differences in symptoms and QoL. Key ME/CFS symptoms (including post-exertional malaise, unrefreshed sleep and neurocognitive impairments) were comparable in presentation between the two illness cohorts. Both ME/CFS and PCC are associated with significant disruptions to life and have an immense and widespread impact on QoL and functioning. This research thereby provides important insight into the presentation and potential prognosis of PCC, serving to guide further development of diagnostic case definitions and care pathways. Additionally, this research foregrounds the illness burdens of ME/CFS and PCC and, consequently, the necessity of accessible multidisciplinary healthcare, disability and social support services for people living with these chronic multi-systemic illnesses. Such patient experiences must be considered in Australian healthcare policy to optimise health outcomes for pwME/CFS and pwPCC in Australia.

## Supplementary Information

Below is the link to the electronic supplementary material.

Supplementary file1 (XLSX 41 KB)

## Data Availability

The datasets generated and analysed during the current study are not publicly available due to confidentiality agreements but are available from the corresponding author upon reasonable request.

## Abbreviations

95% CI: 95% Confidence interval
BMI: Body mass index
C: Consensus
CCC: Canadian Consensus Criteria
CFIDS: Chronic fatigue and immune dysfunction syndrome
HADS: Hospital anxiety and depression scale
HREC: Human research ethics committee
ICC: International Consensus Criteria
M: Median
ME/CFS: Myalgic encephalomyelitis/chronic fatigue syndrome
MFIS: Modified fatigue impact scale
NA: Not applicable
NCNED: National Centre for Neuroimmunology and Emerging Diseases
PCC: Post COVID-19 condition
PROM: Patient-reported outcome measure
PwME/CFS: People with myalgic encephalomyelitis/chronic fatigue syndrome
PwPCC: People with post COVID-19 condition
Q1–3: Quartile 1 to 3
QoL: Quality of life
SARS-CoV-2: Sudden acute respiratory syndrome coronavirus-2
SF-36v2: 36-Item short-form health survey (version 2)
WHO: World Health Organization
WHODAS 2.0: World Health Organization disability assessment schedule (version 2.0)

## Declarations

### Research involving human and/or animal participants

This research has been conducted in accordance with the Griffith University Research Ethics Manual, the Australian Government National Health and Medical Research Council National Statement on Ethical Conduct in Human Research 2007 (updated 2018) [73], and the World Medical Association Declaration of Helsinki [74].
